# Effect of Rare Earth Metals on the Microstructure of Al-Si Based Alloys

**DOI:** 10.3390/ma9010045

**Published:** 2016-01-13

**Authors:** Saleh A. Alkahtani, Emad M. Elgallad, Mahmoud M. Tash, Agnes M. Samuel, Fawzy H. Samuel

**Affiliations:** 1 Industrial Engineering Program, Mechanical Engineering Department, College of Engineering, Prince Sattam bin AbdulAziz University, Al Kharj 11942, Saudi Arabia; salqahtany@hotmail.com (S.A.A.); mahmoud_tash1@yahoo.com (M.M.T.); 2 Département des Sciences Appliquées, Université du Québec à Chicoutimi, Chicoutimi, QC G7H 2B1, Canada; eelgalla@uqac.ca (E.M.E.); amsamuel@uqac.ca (A.M.S.)

**Keywords:** aluminum alloys, La and Ce addition, rare earth metals, modification, intermetallics

## Abstract

The present study was performed on A356 alloy [Al-7 wt %Si 0.0.35 wt %Mg]. To that La and Ce were added individually or combined up to 1.5 wt % each. The results show that these rare earth elements affect only the alloy melting temperature with no marked change in the temperature of Al-Si eutectic precipitation. Additionally, rare earth metals have no modification effect up to 1.5 wt %. In addition, La and Ce tend to react with Sr leading to modification degradation. In order to achieve noticeable modification of eutectic Si particles, the concentration of rare earth metals should exceed 1.5 wt %, which simultaneously results in the precipitation of a fairly large volume fraction of insoluble intermetallics. The precipitation of these complex intermetallics is expected to have a negative effect on the alloy performance.

## 1. Introduction

The discovery of Pacz [1] in 1921, that Al-Si alloys containing between 5% and 15% silicon could be treated with alkali fluoride fluxes (preferably sodium fluoride) to yield alloys of improved ductility and machinability, made it possible to extensively use aluminum foundry alloys in various applications. This treatment is commonly referred to as *eutectic modification* by which the morphology of eutectic silicon may be changed from acicular-like to a finer and more interconnected fibrous structure [2,3].

Chemical modification is the most commonly applied method of modification which produces a fine fibrous silicon structure through the addition of trace levels of certain modifying elements. The modification mechanism induced by such chemical modifiers has been related to the nucleation and growth of Si particles [4,5,6,7]. The modifier addition deactivates the heterogeneous inoculants AlP, oxides, and other melt substrates that act as nucleation sites for the eutectic phase, resulting in an increase of eutectic undercooling and a refined morphology of eutectic silicon. From the point of view of the growth theory, the modifier atoms segregate into favored growth surfaces of the Si crystals, poisoning the attaching mechanism of Si atoms. As a result, the silicon growth is halted and a refined eutectic morphology is obtained.

The most common chemical modifiers include Sr, Na and Sb. Strontium is preferred as a modifying agent in the light of the rapid fading of Na and the toxic effect associated with Sb [2]. The addition of Sr in the range of 150 to 250 ppm was reported to significantly improve the mechanical properties of Al-Si alloys [8,9]. However, if present in larger amounts, Sr can result in the formation of undesirable intermetallic compounds, such as Al_2_SrSi_2_ and Al_4_SrSi_2_, and cause over-modification of the eutectic silicon [10,11].

Rare earth (RE) elements and mischmetal (mixture of RE elements) were also reported to cause modification in both hypo- and hypereutectic Al-Si alloys [12,13,14,15,16,17]. It was found that the modification efficiency of 1.0 wt % La in the microstructures and mechanical properties of A356 alloy is similar to that of 0.01 wt % Sr [12]. Li *et al.* [13] indicated that the addition of 1.0 wt % La or 1.0 wt % Ce to Al-11.7%Si-1.8%Cu alloy could refine the Si particles, but without any obvious modification effect. The effect of different concentrations of individual additions of RE metals including La, Ce, Pr, Nd, Sm, Eu, Gd, Tb, Dy, Ho, Er, Tm, Yb and Lu on the eutectic modification in Al-10%Si was studied [14]. The results showed that all of the RE elements caused a depression of the eutectic growth temperature, but only Eu produced fully-modified, fine-fibrous silicon. The remaining elements resulted in only a small degree of refinement of the plate-like silicon morphology.

Mousavi *et al.* [15] demonstrated that the optimum levels of mischmetal (combination of Ce, La, Pr and Nd) addition to cause modification in A357 Al-Si casting alloy were 0.1 and 0.3 wt % for thin and thick section castings, respectively. The addition of La was reported to optimize the modification effect of P in the Al-18%Si alloy [16]. Zhang *et al.* [17] reported that the addition of Ce to the Al-18%Si alloy changed the primary Si from branched shape to fine facetted shape, and that the modification effect was more obvious for the eutectic Si away from the primary Si. To arrive at a better understanding of the role of RE elements in the eutectic modification, the present study was undertaken to investigate the effect of individual and combined additions of La and Ce in the presence and absence of Sr on the eutectic microstructure of A356 casting alloy. This alloy was specifically selected in this study because it represents one of the most important hypoeutectic Al-Si alloys based on its wide use in the automotive industry which is the largest consumer of Al-Si casting alloys. The modification effect of RE elements was examined using optical and scanning electron microscopy along with thermal analysis technique.

## 2. Experimental Procedures

The chemical compositions of the A356 ingots used in the present study are listed in Table 1, whereas the aimed and actual modifier additions along with the codes of the resulting alloys are listed in Table 2.

**Table 1:** Composition of A356 alloy used in the present study.

Alloy	Elements (wt %)					
	Si	Cu	Mg	Fe	Zn	Al
A356	7	<0.20	0.35	<0.20	<0.10	Bal

**Figure 1:**
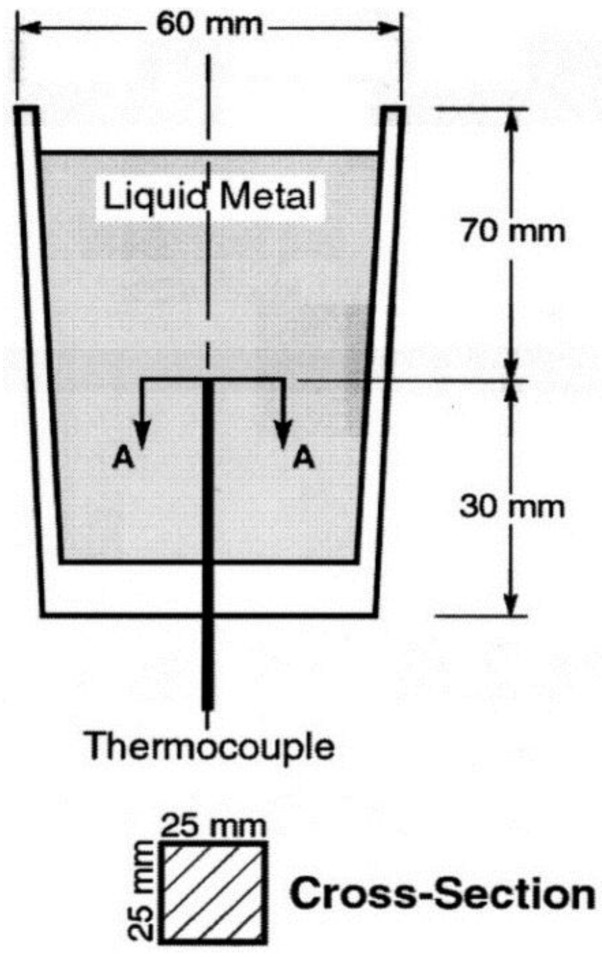
Schematic sketch showing the graphite mold used in this study.

The 12-Kg ingots of A356 alloy were cut into smaller pieces, cleaned, dried, and then melted in a SiC crucible, using an electrical resistance furnace. The melt temperature was kept at 750 ± 5 °C. The holding time before pouring was around 40 s. The modifiers used in the present study, namely Sr, La and Ce, were added in the form of Al-10%Sr, Al-15%La and Al-15%Ce master alloys, respectively. The melt was degassed for 15 min using pure, dry argon injected by using a graphite impeller rotating at 120 rpm. The melt was poured into a graphite mold, a schematic of which is shown in Figure 1. The mold was preheated to 600 °C to obtain cooling rates close to equilibrium conditions. Samplings for spectrochemical analysis were simultaneously taken for each casting poured from the different melts.

Thermal analysis was performed simultaneously using the graphite mold casting setup by attaching a high-sensitive K-type thermocouple to the mold system, passing through the bottom of the mold, and extending half way up into the mold cavity, along the mold centerline. The temperature-time data from the fully liquid state, through the solidification range, to the solid state were recorded using a high-speed PC-based data acquisition system connected to the thermocouple. The part of the thermocouple within the mold was protected using double-walled ceramic tubing. From the thermal analysis data, the cooling curves and their first derivatives were plotted and analyzed.

**Table 2:** The aimed and actual modifier additions and the codes of the resulting alloys.

Alloy	Mold Type	Preheating Mold Temperature (°C)	Alloy Code	Modifier Addition (wt %)					
				Aimed			Actual		
				Sr	La	Ce	Sr	La	Ce
A356	Graphite	600	TB	0	0	0	0	0	0
			TBS	0.01	0	0	0.011	0	0
			T10	0	0.2	0	0	0.17	0
			T1	0	0.5	0	0	0.40	0
			T2	0	1	0	0	0.85	0
			T3	0	1.5	0	0	1.30	0
			T11	0	0	0.2	0	0	0.18
			T4	0	0	0.5	0	0	0.38
			T5	0	0	1	0	0	0.82
			T6	0	0	1.5	0	0	1.38
			T7	0	0.5	0.5	0	0.44	0.38
			T8	0	1	1	0	0.78	0.87
			T9	0	1.5	1.5	0	1.37	1.53
			T10S	0.01	0.2	0	0.009	0.17	0
			T1S	0.01	0.5	0	0.011	0.40	0
			T2S	0.01	1	0	0.008	0.85	0
			T3S	0.01	1.5	0	0.010	1.30	0
			T11S	0.01	0	0.2	0.008	0	0.18
			T4S	0.01	0	0.5	0.009	0	0.38
			T5S	0.01	0	1	0.008	0	0.82
			T6S	0.01	0	1.5	0.010	0	1.38
			T7S	0.01	0.5	0.5	0.009	0.44	0.38
			T8S	0.01	1	1	0.009	0.78	0.87
			T9S	0.01	1.5	1.5	0.011	1.37	1.53

TB: Base A356 alloy; TBS: Sr-modified base A356 alloy.

For metallographic observations, samples were sectioned from each casting, mounted, and then polished. The polished samples were examined using an optical microscope and a scanning electron microscope (Hitachi-SU-8000, Hitachi High-Technologies Corporation, Tokyo, Japan). The quantitative analysis of the silicon particle characteristics was carried out using a Clemex image analyzer system (Clemex Technologies Inc., Longueuil, QC, Canada) in conjunction with the optical microscope. In each case, the measurements were carried out over fifty fields at 200× magnification such that the entire sample surface was traversed in a regular, systematic fashion, to obtain the average values of the measured parameters.

## 3. Results and Discussion

### 3.1. Thermal Analysis

**Figure 2:**
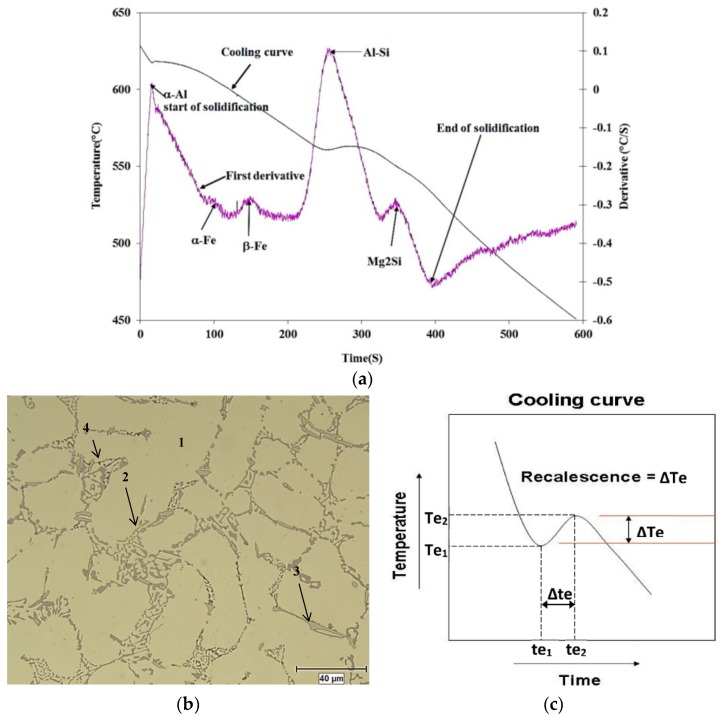
(**a**) Solidification curve and its first derivative of A356 alloy; (**b**) Optical microstructure of A356 alloy (coded TB) following solidification at the rate of ~0.8 °C/s: 1—α-Al, 2—eutectic Si, 3—Fe-intermetallic, 4—Mg_2_Si phase; (**c**) Schematic diagram of a hypothetical cooling curve showing recalescence.

The solidification curve for the base alloy, obtained from its time-temperature data, and its first derivative plot are provided in Figure 2a. The temperatures at which the main reactions take place during solidification were determined from the first derivative curve and are marked on the diagram. Figure 2b displays the micro-constituents of the microstructure after solidification at the rate of approximately 0.8 °C/s.

Figure 2c shows a diagram representing recalescence (Δ*T*) which is defined as the heat generated during solidification relating to the nucleation and growth of a new phase [18]. Without intending to make an in-depth approach to the mathematics of solidification, but rather to understand the constituent parts governing the solidification process itself, Equation (1) provides a basic mathematical formula which introduces the principal parameters of recalescence:

**Δ*T* = [(Δ*hf*/*cp*)(δ*fs*/δ*t*)] − [(*q_e_*/*cp*)(*A*/*V*)]** (1)

As will be observed from Equation (1), **Δ*hf*** is the latent heat of fusion denoting a decrease in enthalpy due to the transformation from liquid to solid; ***cp*** is the specific heat per unit volume; ***q_e_*** is the heat flux; ***A*** and ***V*** are area and volume, respectively, of the solidifying sample; and ***fs*** is the solid fraction. The first term on the right-hand side of Equation (1) takes into account the continuing evolution of the latent heat of fusion during solidification. The second term reflects principally the effects of casting geometry, area, and volume of the solidifying samples. Regarding this equation, recalescence will occur when the first term on the right-hand side of Equation (1) becomes greater than the second one.

As may be deduced from Equation (1), the heat extraction of the samples in thermal analysis plays an essential role in perceiving recalescence based on solidification data, in the eventuality that there is a high discrepancy between both terms of this equation; for example, if the second term is much higher than the first term, the perception of the recalescence may remain concealed, and as a result, the thermal analysis data will not reveal any noticeable reaction. Generally speaking, throughout the course of thermal analysis experiments at slow cooling rates, the heat loss from the surrounding environment exceeds the heat generated by nascent, or incipient, phase reactions to a great extent, specifically during the initial solidification period, making it impossible to detect their appearance on the cooling curve, *i.e.*, to perceive the recalescence [19].

**Figure 3:**
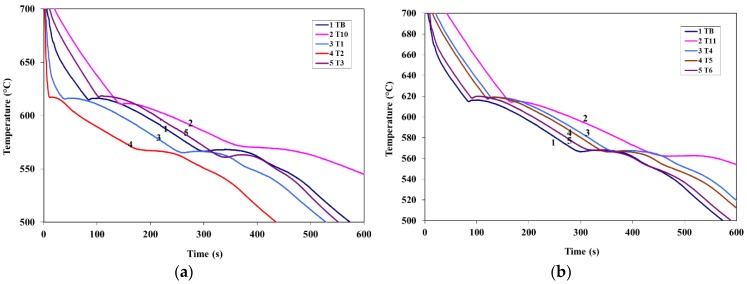
(**a**) Solidification curves of La-containing alloys; (**b**) solidification curves of Ce-containing alloys.

**Figure 4:**
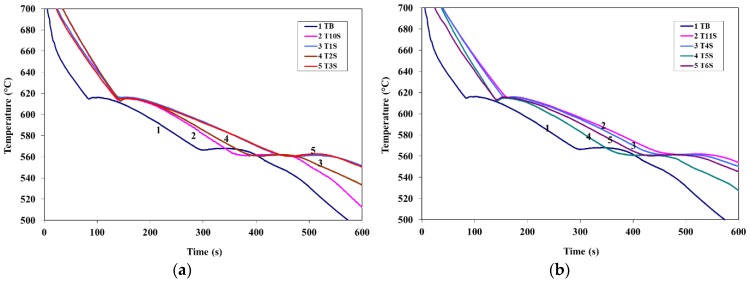
(**a**) Solidification curves of La+Sr-containing alloys; (**b**) solidification curves of Ce+Sr-containing alloys.

Figure 3 and Figure 4 show the solidification curves obtained from the present alloys with compositions listed in Table 2, whereas Table 3 and Table 4 list the variation in the eutectic recalescence in alloys containing La (as an example). It is evident from these data that;

1. Introduction of La or Ce increased the start of solidification temperature of A356 alloy by about 11 °C at 1.5 wt % La or Ce with a marginal effect on the Al-Si eutectic temperature.
2. Introduction of 100 ppm Sr to A356 alloy reduced the eutectic temperature by about 7 °C. Addition of La, Ce, or La + Ce up to 1.5 wt % each has no further effect on the eutectic temperature.
3. No explicit peak corresponding to precipitation of rare earth (RE)—containing phases could be detected using the thermal analysis technique.
4. Although the introduction of Sr has no noticeable effect on the Al-Si eutectic recalescence temperature (Δ*T*), the recalescence time increased from 20 s to 40 s as inferred from Table 3 and Table 4. In this case as well, the presence of La or Ce did not have an effect on either Δ*T* or Δ*t*.

**Table 3:** Effect of La addition on the recalescence of the eutectic Si.

Alloy Code	Recalescence of Eutectic Si					
	*Te*_1_ *	*Te*_2_	Δ*Te*	*te*_1_ **	*te*_2_	Δ*te*
T10	565.1	566.2	1.1	242.5	252.3	9.8
T1	565.2	566.4	1.2	239.8	260.2	20.4
T2	563.4	564.8	1.4	254.3	270.3	16.0
T3	560.8	563.2	2.4	257.0	281.2	24.2

* Temperature (°C); ** time (s).

**Table 4:** Effect of La and Sr additions on the recalescence of the eutectic Si.

Alloy Code	Recalescence of Eutectic Si					
	*Te*_1_	*Te*_2_	Δ*Te*	*te*_1_	*te*_2_	Δ*te*
T10S	560.7	562.6	1.9	264.6	308	43.4
T1S	560.4	561.8	1.4	339.6	388.8	49.2
T2S	560.6	562.2	1.6	279.6	322.2	42.6
T3S	560.7	563.0	2.3	347.8	389.4	41.6

### 3.2. Microstructural Characterization

**Figure 5:**
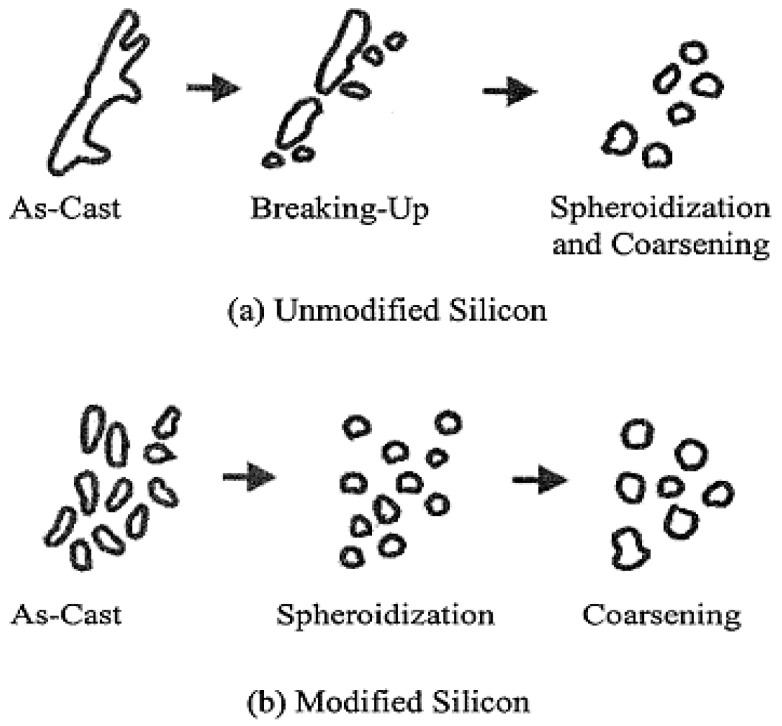
Schematic characterization of the three stages of spheroidization and coarsening of the eutectic silicon phase in the case of (**a**) unmodified; and (**b**) modified Al-Si alloy [20].

The coarsening process of eutectic Si particles, called Ostwald ripening, holds that larger particles grow at the expense of smaller ones. Solution treatment tends to spheroidize constituents which cannot be fully dissolved, as in the case of casting Al-Si alloys. At the beginning of the solution treatment, the acicular silicon platelets in the unmodified structure begin to break down into smaller fragments and gradually spheroidize. In modified structures the spheroidization takes place at an early stage [20]. Figure 5 shows a schematic representation of the spheroidization and coarsening process.

**Figure 6:**
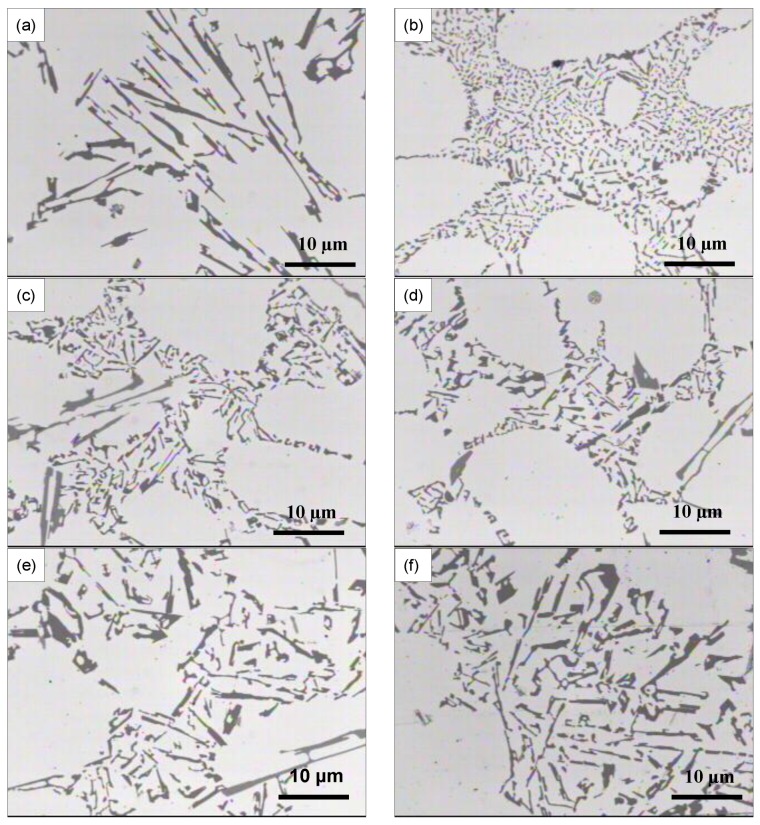
Microstructures of different A356-based alloys: (**a**) base TB alloy (non-modified); (**b**) TBS alloy (0.01% Sr); (**c**) T10 alloy (0.2% La); (**d**) T11 alloy (0.2% Ce); (**e**) T2 alloy (1% La); and (**f**) T5 alloy (1% Ce).

Figure 6 show the microstructure of the A356 alloy in the non-modified and different modified conditions. In the non-modified condition (Figure 6a), the eutectic silicon typically displayed a coarse plate-like structure. The addition of 0.01% Sr (Figure 6b) significantly modified the eutectic silicon, changing its morphology from a coarse plate-like structure to a fine fibrous one. The addition of 0.2% La or 0.2% Ce (Figure 6c,d, respectively) resulted in a relative refinement in the plate-like silicon morphology when compared to the non-modified TB alloy (Figure 6a), but without any obvious modification effect. Such a refining effect of La and Ce was also reported by other researchers [13,14] for eutectic and hypoeutectic Al-Si alloys, respectively. However, neither refining nor modifying effect was observed for La or Ce additions beyond 0.2%, *i.e.*, 0.5% and 1%. This can be discerned from the micrographs shown in Figure 6e,f, which were obtained from A356 alloy samples modified by 1% La and 1% Ce, respectively. It is interesting to note that the addition of 1% La or 1% Ce caused a noticeable coarsening of eutectic silicon particles. It was also observed that the combined addition of 0.5% La and 0.5% Ce or 1% La and 1% Ce did not bring about any substantial change in the eutectic silicon morphology.

**Figure 7:**
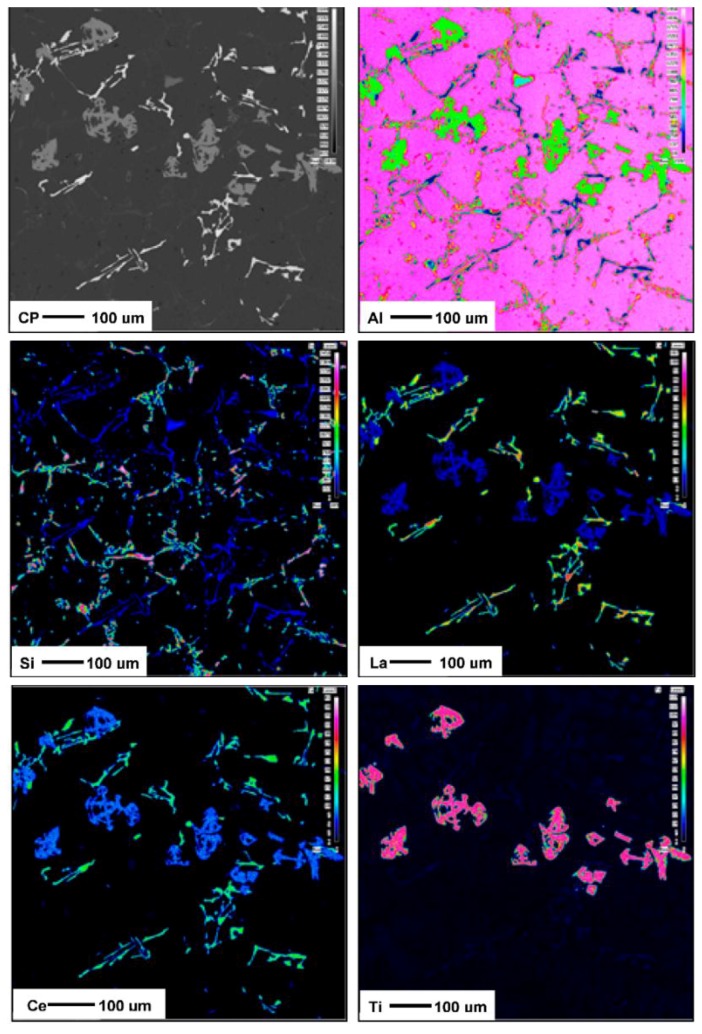
Backscattered electron (BSE) images showing the formation of La- and Ce-rich phases in an A356 alloy sample modified by the combination of 1% La and 1% Ce (T8 alloy) and the corresponding *X*-ray images of Al, Si, La, Ce and Ti.

**Figure 8:**
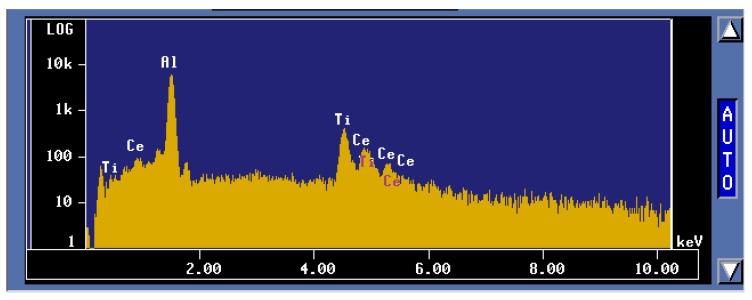
EDS spectrum corresponding to the gray phase observed in the BSE image in Figure 7 (CP).

**Figure 9:**
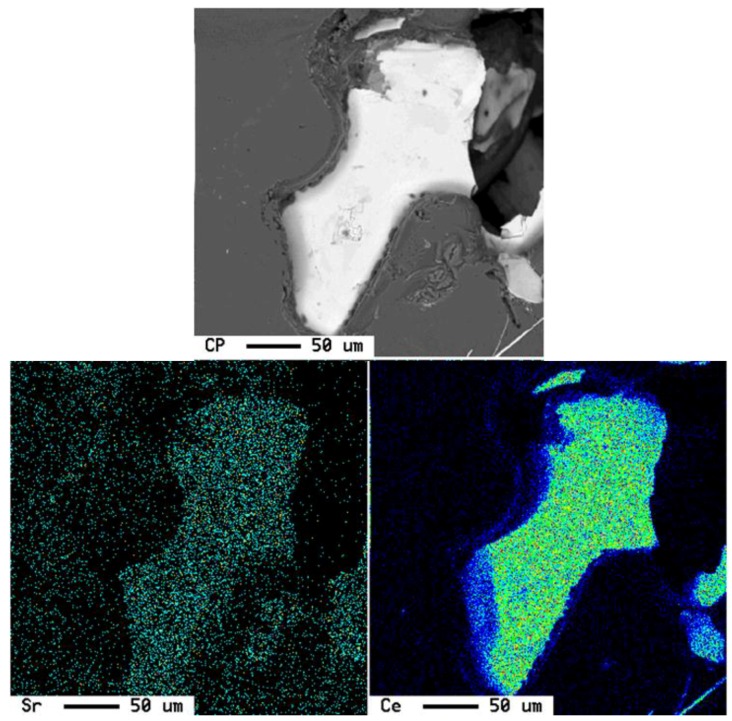
Ce-Sr interactions in A356 alloy modified with 1.0% Ce + 0.01% Sr (T5S alloy).

**Figure 10:**
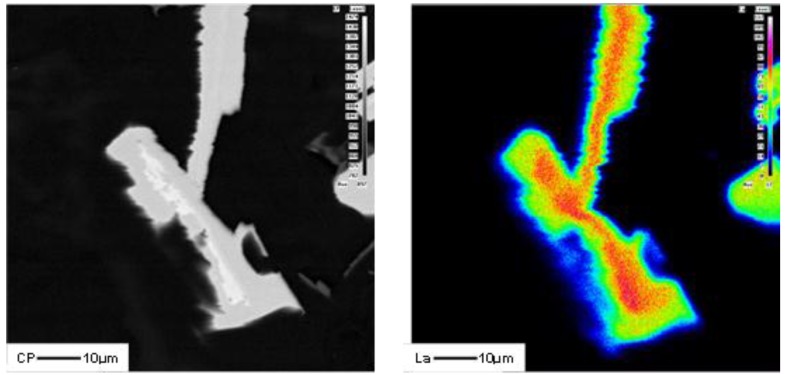

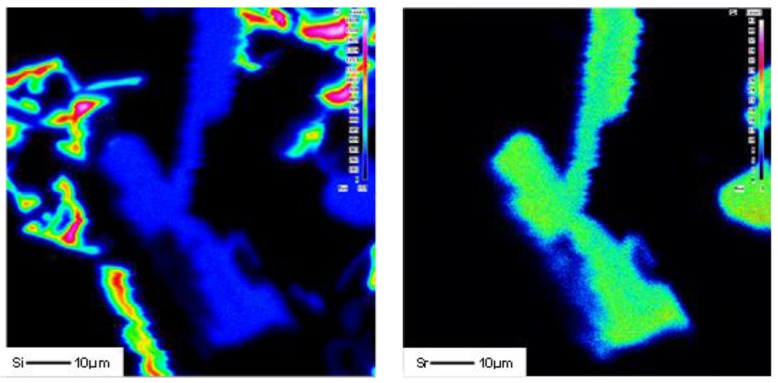
La, Si and Sr distribution in A356 alloy modified with 1.0% La + 0.01% Sr (T2S alloy).

**Figure 11:**
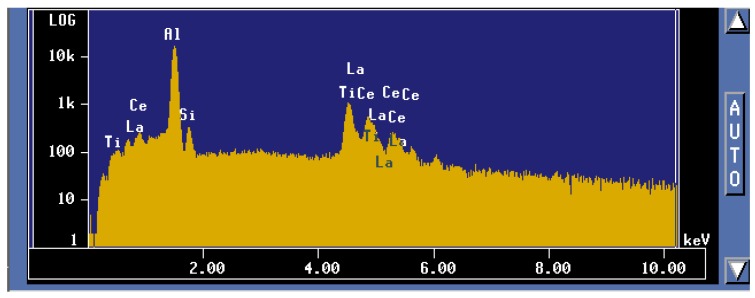
EDS spectrum corresponding to Figure 10, revealing a significant peak due to Si.

To better understand these observations, *X*-ray mapping of intermetallic phases found in an A356 alloy sample modified by the combination of 1% La and 1% Ce (T8 alloy) was carried out to determine the distribution of these elements, as shown in Figure 7. The corresponding enregy dispersive *X*-ray spectroscopic [EDS] spectra are shown in Figure 8. It is evident that the addition of La and Ce resulted in the formation of two intermetallic phases which appeared as coarse white and gray particles in the backscattered image shown in Figure 7. In addition to La and Ce, these phase also contained different contents of Al, Si and Ti. It can therefore be suggested that high levels of La and/or Ce can result in the formation of coarse intermetallic compounds instead of modifying or even refining the eutectic silicon.The formation of La- and Ce-rich intermetallic phases in Al-Si alloys was also reported in several studies [15,21,22]. A new AlSiLa intermetallic phase was detected in an A357 alloy when increasing the La-based mischmetal level beyond 0.3% [15]. Elsebaie *et al.* [21], and Hosseinifar and Malakhov [22] reported the formation of complex phases such as AlTiLa(Ce)Mg, AlSiLa(Ce) and La(Al,Si)_2_ phases. Figure 9 and Figure 10 display the affinity of RE metals to react with Sr leading to the observed loss of modification in the present alloys. Moreover, La/Ce revealed a clear tendency to react with Si forming a complex intermetallic as shown in Figure 11, which is in good agreement with the abovementioned results [21,22].

**Figure 12:**
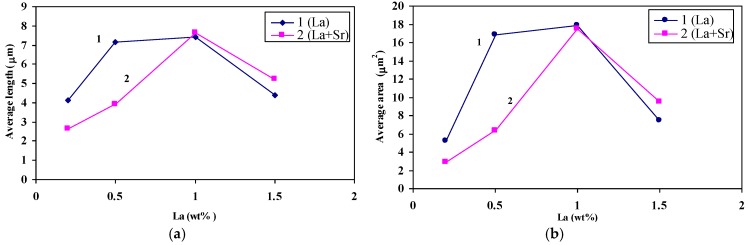
(**a**) The effect of La and Sr on the average of Si particle length; (**b**) the effect of La and Sr on the average of Si particle area.

**Figure 13:**
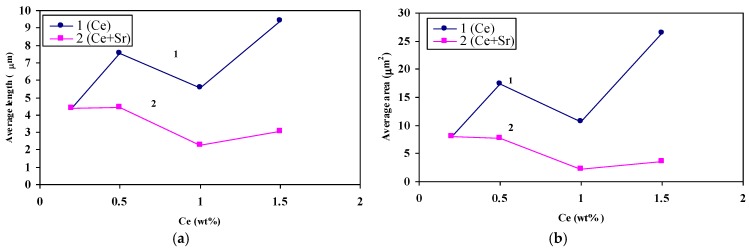
(**a**) The effect of Ce and Sr on the average of Si particle length; (**b**) the effect of Ce and Sr on the average of Si particle area.

**Figure 14:**
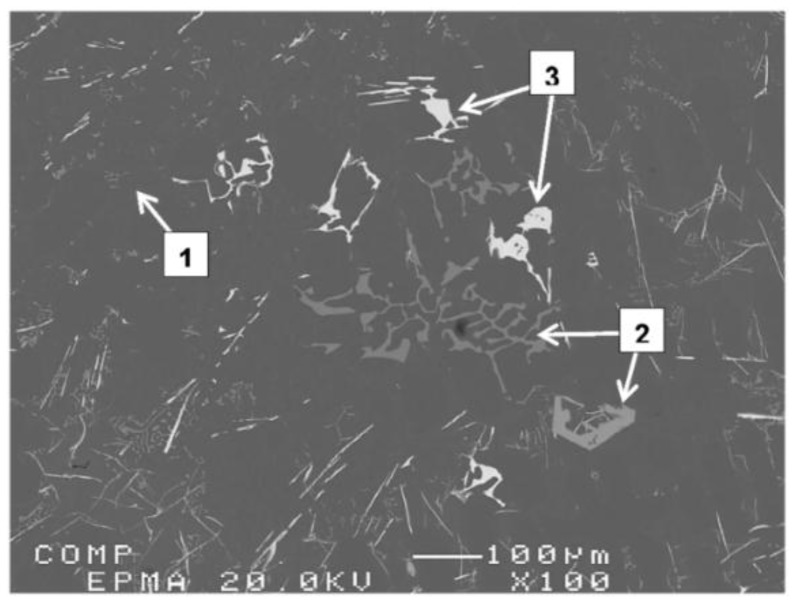
Precipitation of La-rich phases in A356 alloy containing 1.5 wt % La (T3 alloy): (1) α-Al; (2) α-Fe and (3) La-rich phase.

Figure 12 and Figure 13 illustrate the variation in Si particle characteristics as a function of the added La or Ce, respectively. It is evident from these diagrams that the affinity of La to react with Sr leading to degradation of modification effectiveness is greater than that of Ce. This observation is limited to 1 wt % of La beyond which a dramatic decrease in the Si particle size is seen to take place (Figure 12). The observed decrease in the size of the eutectic Si particles may interpreted in terms of La-Si interaction forming AlSiLa and La(Al,Si)_2_ compounds resulting in significant reduction in the amount of free Si. Figure 14 shows the precipitation of insoluble La-rich intermetallics in A356 alloy containing 1.5 wt % La (coded T3 in Table 2). Similar findings have been reported by Hong-Kun and Di [23] who added 3 wt % of La to Al-17 wt %Si. However, the presence of such hard brittle intermetallics would have a negative effect on the alloy mechanical properties [24].

## 4. Conclusions

Based on the results documented in the present work, the following conclusions may be drawn:

1. Addition of La or Ce individually or combined up to 1.5 wt % increases the melting point of A356 alloy at approximately 1 °C/0.15 wt % RE metals.
2. The addition of RE metals has no significant effect either on the Al-Si eutectic temperature or on the modification of the eutectic Si particles.
3. Both La and Ce have high affinity to react with Sr resulting in marked reduction in the Sr modification effect.
4. Modification of Si particles may take place when La concertation exceeds 1.5 wt %.
5. Increasing the amount of La, however, leads to precipitation of insoluble intermetallics that could negatively affect the alloy performance.
